# Prolonged incubation of severe acute respiratory syndrome coronavirus 2 (SARS-CoV-2) in a patient on rituximab therapy

**DOI:** 10.1017/ice.2020.1239

**Published:** 2020-10-07

**Authors:** Alan G. Koff, Maudry Laurent-Rolle, Jack Chun-Chieh Hsu, Maricar Malinis

**Affiliations:** 1 Section of Infectious Diseases, Department of Internal Medicine, University of California Davis, Davis, California; 2 Section of Infectious Diseases, Department of Internal Medicine, Yale University School of Medicine, New Haven, Connecticut; 3 Departments of Immunobiology and Cell Biology, Yale University School of Medicine, New Haven, Connecticut

## Abstract

The incubation period of severe acute respiratory syndrome coronavirus 2 (SARS-CoV-2) is rarely >14 days. We report a patient with hypogammaglobulinemia who developed coronavirus disease 2019 (COVID-19) with a confirmed incubation period of at least 21 days. These findings raise concern for a prolonged presymptomatic transmission phase, necessitating a longer quarantine duration in this patient population.

Severe acute respiratory syndrome coronavirus 2 (SARS-CoV-2) was discovered in Wuhan, China, and has since become a global pandemic through person-to-person spread. SARS-CoV-2 exhibits presymptomatic transmission during the incubation period, where an individual is contagious prior to symptom onset.^1^ Defining the incubation period, therefore, has infection control and public health implications because a longer incubation necessitates a longer quarantine duration after an exposure.

Mean incubation periods range from 5.0 to 7.2 days, and a median incubation period of 5.1 days has been reported.^2–6^ In 2 studies, the 95th percentiles of the distribution were reported as 12.5 days and 13 days, and another 3 studies reported the 99th percentile as 11.9 days, 14 days, and 14.9 days, respectively.^2–4,6,7^ In the vast majority of cases, the incubation period is far less than 14 days, which has helped to inform the Centers for Disease Control and Prevention (CDC) recommendations for a 14-day quarantine period after a known coronavirus disease 2019 (COVID-19) exposure.^8^ However, these cases represent the general population and do not provide detailed information on subpopulations in whom the incubation period may differ. Herein, we present a case with objectively confirmed COVID-19 with a prolonged incubation period proven through viral culture.

## Case presentation

A 71-year-old female on rituximab for granulomatosis with polyangiitis presented with shortness of breath and nonproductive cough. Six weeks prior to admission, several family members had been diagnosed with COVID-19 infection, prompting her to undergo testing despite being asymptomatic. Her nasopharyngeal (NP) swab polymerase chain reaction (PCR) test for SARS-CoV-2 was positive. She was self-isolating, and her only contact was a family member who had recovered from mild COVID-19 illness and had since been asymptomatic. Repeat NP PCR testing 13 days later was also positive. On day 21 after the first test, the patient developed progressive dyspnea on exertion, a minimally productive cough, significant fatigue, and nonbloody diarrhea.

She was admitted to hospital on day 36 after her first test. She was febrile to 38.8°C and her oxygen saturation was 93% on room air. She was placed on 2 L/minute of supplemental oxygen. Computed tomography (CT) of the chest demonstrated bilateral peribronchovascular ground-glass opacities (Supplementary Fig. 1 online). Relative to the day of her first test, she had repeat SARS-CoV-2 NP PCR tests on days 36, 37, and 40, which were negative. Serology for SARS-CoV-2 was negative. Flow cytometry of peripheral blood demonstrated no circulating B-cells, and an immunoglobulin panel demonstrated low levels of IgM, IgG, and IgA consistent with a history of receiving rituximab. Bronchoalveolar lavage (BAL) on hospital day 5 revealed a positive SARS-CoV-2 PCR with N1 and N2 cycle thresholds of 29 and 28, respectively. The patient was weaned off supplemental oxygen and was discharged on hospital day 9.

**Fig. 1.**
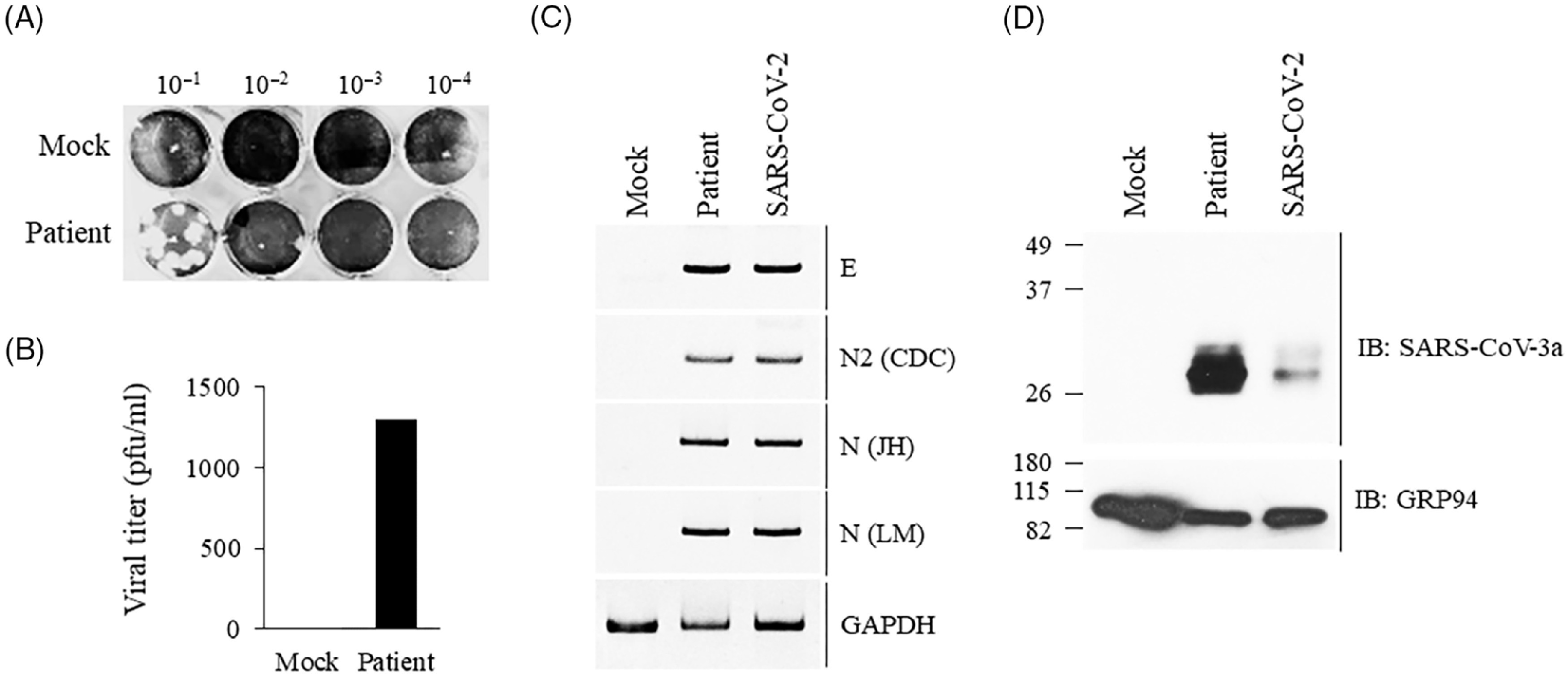
Persistent SARS-CoV-2 viral replication in an immunocompromised patient. Vero E6 cells were mock-infected or inoculated with patient’s bronchoalveolar lavage fluid for 4 days. Viral supernatants from passage 1 were collected and used for plaque assay using Vero E6 cells. Representative plaque assay shown (1A). Plaques counted to deduce viral titer as plaque-forming units (1B). Cell lysate used for qRT-PCR. SARS-CoV-2 virus (2019-nCoV/USA_WA1/2020) was used as a positive control (1C). Vero E6 cells were mock-infected or infected with virus isolated from passage 1 or a control SARS-CoV-2 virus (2019-nCoV/USA_WA1/2020) for 48 hours followed by immunoblot analysis of SARS-CoV-2 3a protein using an antibody against SARS-CoV 3a (1D). The primer sequences can be found in Supplementary Table 1 (online).

The patient’s BAL fluid was stored at −80°C then thawed and inoculated into Vero E6 cell culture. Viral supernatant was harvested on day 4 after inoculation for plaque assay demonstrating infectious virus with a titer of 1.3 × 10^3^ pfu/mL on passage 1 (Fig. 1A and 1B). Nucleic acid extraction from the cell lysate confirmed the presence of SARS-CoV-2 by reverse-transcription real-time PCR and by polyacrylamide gel (Fig. 1C). Isolate from the first passage of the BAL specimen was used to infect Vero E6 cells for 48 hours. Cell lysates were probed for protein analysis using an antibody raised against SARS-CoV 3a antibody which demonstrated bands consistent with SARS-CoV-2 3a protein (Fig. 1D). These studies indicate that infectious SARS-CoV-2 virus was isolated from the patient’s BAL.

## Discussion

This case demonstrates an objectively confirmed asymptomatic SARS-CoV-2 infection with symptom onset 21 days after her positive test. Furthermore, since an NP PCR can be falsely negative on the first day of infection, her incubation period may have been even greater.^9^ Lower respiratory tract sampling demonstrated viable SARS-CoV-2 virus, though the NP PCR was negative. A prior study demonstrated that NP PCR had a false negative rate of 66% by day 21, which may explain our observation.^9^

Reports of incubation periods >21 days are very rare. A patient with an incubation period of 24 days was reported; however, the incubation period was defined as the time between the earliest potential date of exposure to the first day of symptom onset, potentially leading to overestimation.^6^ A case report described a patient with an incubation period of at least 38 days based on a social history of limited contact with others after an exposure.^10^ Whether our patient’s absence of circulating B cells with subsequent hypogammaglobulinemia predisposed her to a prolonged incubation period is not known. Her negative serology suggests a poor humoral response to infection.

This report has significant implications for preventing the spread of SARS-CoV-2. For patients with known humoral immune deficits, until further data are available, one should exercise caution using a 14-day quarantine window based on the assumption of 14 days being the upper bound of the incubation period. It remains possible that this patient was shedding viable virus from the date of her initial positive test to beyond the date of her bronchoscopy 41 days later. This patient’s presymptomatic transmission window may have therefore been substantially greater than the estimated mean presymptomatic transmission window of 2.3 days in the general population.^1^ Whether prolonged incubation periods may occur in other immunosuppressing conditions remains to be evaluated, and further data in this area are needed to better define the appropriate quarantine period in this population.

## Supplementary material

For supplementary material accompanying this paper visit https://doi.org/10.1017/ice.2020.1239. click here to view supplementary material
