# Supplementary material for: Prolonged incubation of severe acute respiratory syndrome coronavirus 2 (SARS-CoV-2) in a patient on rituximab therapy
Source: Infect Control Hosp Epidemiol. 2020 Oct 7:1–2. doi: 10.1017/ice.2020.1239 (PMC7578652; doi:10.1017/ice.2020.1239)
Supplement: Supplementary file 1 [file S0899823X20012398sup.zip › S0899823X20012398sup002.docx]

**Supplementary Fig. 1.** Computed tomography of the chest with intravenous contrast**.** Bilateral peribronchovascular groundglass opacities are noted consistent with COVID-19.
